# Pyrogeographic zonation: Implications for fire management at the local level

**DOI:** 10.1371/journal.pone.0328233

**Published:** 2025-08-04

**Authors:** Christoph Neger, Camila Toledo-Jaime, Leopoldo Galicia, Octavio Romero-Cuapio

**Affiliations:** 1 Academic Unit for Territorial Studies Yucatán, Institute of Geography, National Autonomous University of Mexico, Mérida, Mexico; 2 Postgraduate Program in Geography, National Autonomous University of Mexico, Mexico City, Mexico; 3 Department of Physical Geography, Institute of Geography, National Autonomous University of Mexico, Mexico City, Mexico; 4 Postgraduate Program in Geography, National Autonomous University of Mexico, Mexico City, Mexico; State University of Feira de Santana: Universidade Estadual de Feira de Santana, BRAZIL

## Abstract

Fire management needs to consider the concept of pyrogeography, which highlights the coincidence of different human and natural factors that result in the fire incidence patterns found in a landscape. Recently, several studies have taken this concept further to define regions at the continental, macro-regional or national levels. However, our understanding of this concept is limited at the landscape scale. The present paper aims to adapt this approach at the local level via the hierarchical clustering of fire-related data applied to the La Sepultura biosphere reserve, the protected area with the highest wildfire incidence in Southern Mexico. The resulting clusters were not wholly contiguous yet show a specific spatial distribution. The paper relates these clusters to the spatial configuration of fire management activities in the area. It discusses the usefulness of pyrogeographic zones regarding the strategic planning of fire management activities.

## Introduction

Wildfire incidence and behaviour closely relate to factors of the physical environment, like climatic factors, topography, and vegetation, which determine the availability and characteristics of fuel loads [1,2]. However, for thousands of years, humans have also shaped fire occurrence; today, most wildfire causes are directly related to human activities [3,4]. Together, these human and physical factors form a region’s pyrogeography, a concept that embraces the full array of factors that influence fire's role in the landscape [1,5,6]. Several recent studies have further developed this concept, defining pyrogeographic regionalization as a guide to adapt fire management to the specific spatial conditions of different areas.

Table 1 gives an overview of previous studies conducted in this regard. The first study mentioned by Moreno and Chuvieco, from 2013, does not yet consider a holistic perspective of the areas' pyrogeography as it only considers fire data [7]. However, it is an essential forerunner for this kind of analysis, including advances such as the analysis based on a grid, given the differences in the size of other potential spatial units such as municipalities. Also, the study of Boulanger and his co-authors, published the same year, still was not mainly focused on defining pyroregions but rather on projecting the potential impact of climate change on fire regimes on a 60x60 km grid, considering a wide array of climate data; it also included three other environmental metrics as well and one human factor (road density) [8].

**Table 1:** Overview of previous studies regarding de definition of pyrogeographic zones.

Authors	Year	Study area	Spatial units	Variables	Method	Results
Moreno & Chuvieco	2013	Spain	10x10 km grid	Fire regime metrics (6)	Different statistical analysis to obtain uncorrelated data, clustering based on k-means algorithm	4 clusters
Boulanger et al.	2013	Eastern Canada	60x60 km grid	Fire regime (4), climate (14), environmental (3) and socio-economic (1) metrics	Random Forests modeling	Different zonations of current and future fire regimes in a changing climate
Wu et al.	2015	Boreal forests of northeastern China	Not clearly defined, based on point data and raster with varying cell sizes	Fire regime (4), climate (2), environmental (4) and human (3) metrics	Kernel density estimation and ArcGIS Grouping analysis	3 zones
Trigo et al., Calheiros et al.	2016, 2020, 2021	Iberian Peninsula	Administrative regions	Fire regime (1)	k-means clustering	4 zones
Conedera et al.	2018	European Alps	NUTS3 regions	Fire regime (7), climate (7), environmental (4) and socio-economic (3) metrics	Hierarchical clustering based on Bray-Curtis dissimilarity	3 main clusters and 2 outlier clusters
Elia et al.	2022	Italy	NUTS3 regions	Fire regime (8), climate (4), environmental (17) and socio-economic (5) metrics	Clustering based on the affinity propagation algorithm	7 clusters
Resco de Dios et al.	2023	European Mediterranean	Ecoregions	Fire regime (2)	k-means clustering	5 clusters
Resco de Dios et al.	2023	China	Ecoregions	Fire regime (1)	k-means clustering	5 clusters
Galizia et al.	2023	Europe	50 x 50 km grid	Fire regime metrics (5) and Fire Weather Index	Cluster analysis (no detailed explanation)	5 clusters
Cunningham et al.	2024	Australia	Delineation based on Gaussian mixture models	14 fire regime metrics derived from 5 data sources	Random Forests modelling	17 clusters

The first attempt to precisely define zones for fire management based on the clustering of pyrogeographic variables (defined as “fire environment zones”) was undertaken by Wu and collaborators in 2015, resulting in three contiguous and, within themselves, rather heterogeneous zones [9]. On the contrary, a year later, Trigo and his colleagues defined four contiguous and consistent clusters in the Iberian Peninsula based only on data from burned areas [10]. Then, they used the resulting clusters for more advanced analysis regarding meteorological influences, an approach which was later further developed by other authors [11,12].

A study by Conedera and collaborators stands out for the variety of variables used, given the optimal availability of data in Europe [5]. Their analysis resulted in three clearly defined and spatially mostly contiguous clusters and two outlier clusters. As they argue, these clusters may represent a crucial baseline for detecting shifts in regional fire regimes in the future. A similar study with a combination of an even higher number of different variables was carried out by Elia and other colleagues, resulting in seven clusters, which were mostly contiguous but had a few outliers [6].

Other recent studies have focused more intensely on climate and pyroregions. Resco de Dios and his coauthors used only one or two fire metrics, respectively, combined with a classification of ecoregions, to define the pyroregions and then went on to perform a more complex analysis of the impacts of weather on fire based on the resulting clusters [13,14]. More recent studies focused on combining different fire regime metrics and using the results to create projections on future fire regime changes [15,16].

The present paper sets out from the hypothesis that the approach to defining pyrogeographic zones also applies locally, within a region or a protected area. This relates to the often rich diversity of ecosystems and topographic units within small areas, particularly mountainous regions. La Sepultura Biosphere Reserve in Southern Mexico, which contains a variety of ecosystems adapted to and sensitive to fire [17], is taken here as a model to study down-scaling the pyrogeographic zonation. Furthermore, it is the protected area with the highest fire incidence in Southern Mexico (according to data provided by the National Commission of Protected Natural Areas) and is part of one of Mexico’s main fire clusters [18].

Following particularly the holistic approach laid out in the work of Conedera, Elia and their co-authors [5,6], the study revises potential variables to consider and revises their correlation and collinearity before integrating them into a cluster analysis to identify zones related to the area´s pyrogeography. Subsequently, the study discusses these results and compares them to the current fire management arrangements in the area according to governmental data. Moreover, it discusses how the results of this study might be helpful in orienting fire management strategies.

## Materials and methods

### Study area

**Fig 1:**
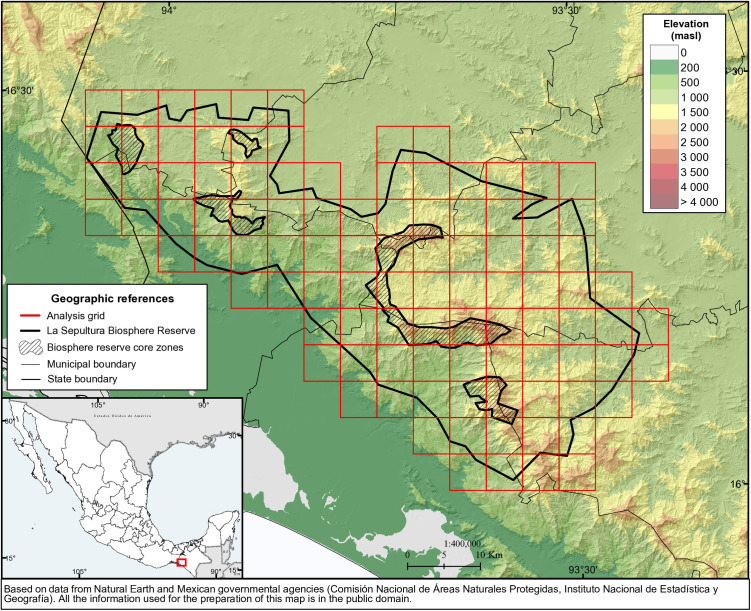
Location of the study area and application of a 5 x 5 km grid (data derived from Natural Earth and Mexican governmental agencies [20–23]; all information in the figure is in the public domain).

The La Sepultura biosphere reserve, established in 1995 in Chiapas, Mexico, has a surface area of 1,673 km^2^, divided into a buffer zone and several smaller core zones, which in total make up 8.2% of the reserve's area [19] (see the map in Fig 1 based on data from Natural Earth and Mexican governmental agencies [20–23]). The reserve belongs to the *sierras del sur de Chiapas*, a mountain range of granite rocks [24], with a maximum elevation within the reserve of 2550 m [19]. This topography and the area's varied micro-climates give rise to a diverse mosaic of different types of vegetation, including fire-prone pine and oak forests as well as fire-sensitive cloud forests and tropical dry forests [17], which are home to a rich biodiversity [19].

While the core zones are strictly protected, human settlements and low-impact activities such as forestry, extensive agriculture and animal husbandry are permitted in the buffer zone [19]. The population living within the reserve in 2020 was 9843 inhabitants; however, when considering communities within its proximity and whose population owns land within the reserve, the number rises to more than 25 thousand (own calculations based on official statistical data [21,25]).

An outstanding feature of the reserve is the high annual wildfire incidence. This is related in part to the presence of fire-prone vegetation and, in part, to fire use by the local population [17]. Therefore, this area has been identified as the centre of one of the four main fire clusters in Mexico, southern Chiapas, the only one in the South of the country, characterized by a short fire season with an early peak in April, and reaching into May [18].

### Data and analysis

#### Data acquisition and definition of the study units.

**Fig 2:**
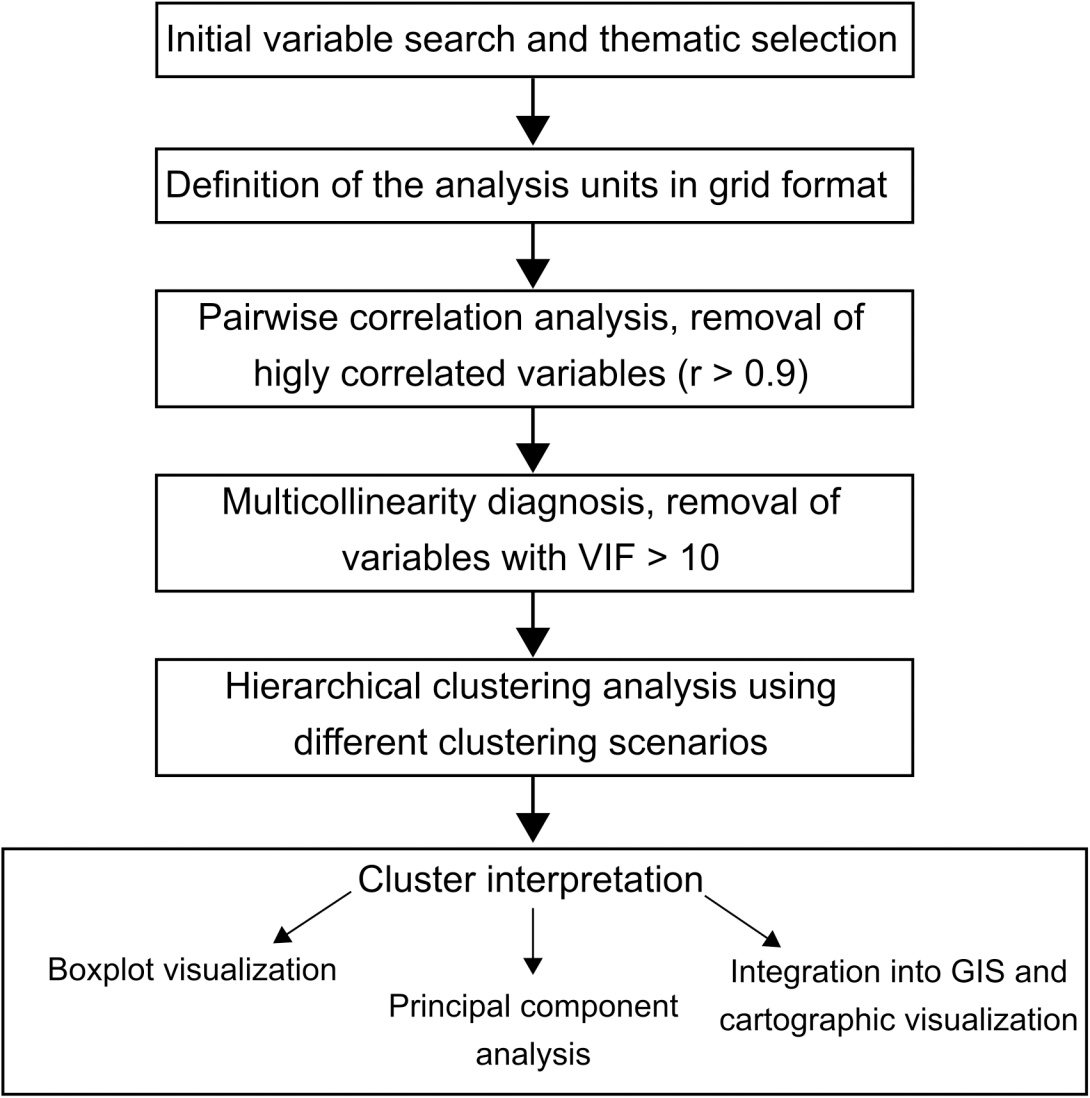
Methodological flowchart of the study’s approach to pyrogeographic clustering.

Fig 2 gives an overview of the methodological process of this work. The search and primary data selection considered two main criteria: first, the kinds of data used in previous pyrogeographic regionalizations as outlined in the introduction, and second, the data availability from governmental institutions in Mexico (Table 2). General data on the area's human and physical geography came from the National Institute of Statistics and Geography (www.inegi.org.mx) and the geoportal of the National Commission for the Use and Conservation of Biodiversity (http://geoportal.conabio.gob.mx/). For detailed fire data, the study consulted the information available in the national forest information system provided by the National Forestry Commission (https://snif.cnf.gob.mx/incendios/).

**Table 2:** Variables considered in the analysis.

Category	Variable	Code	Units
**Biosphere reserve boundaries**	Buffer zone	BZ_area	km^2^
	Core zone	CZ_area	km^2^
**Physical environment**	Forest cover	Forest_Cov	km^2^
	Fire-adapted vegetation	Veg_Adapt	km^2^
	Vegetation sensible to fire	Veg_Sens	km^2^
	Water bodies	Water	km^2^
	Average terrain slope	Slope	degrees
	South-facing slope	South_Slope	km2
	Average annual precipitation	Precip	mm
	Average annual temperature	Temp	degrees C
	Average elevation	Elev	m
**Population**	Total population	Pob_Tot	individuals
	Population speaking an indigenous language	PSIL	individuals
	People with primary education completed	PPEC	individuals
	Number of human settlements	N_Sett	occurrences
	Area covered by human settlements	A_Sett	km^2^
	Area covered by crops	Crop_Cov	km^2^
	Highway kilometers	Hwy_Km	km
**Fire data**	Number of fires	N_fires	occurrences
	Total burned area	BA_Tot	km^2^
	Ratio burned area/ forest cover	BA/FC	ratio
	Ratio number of fires/ forest cover	NF/FC	
	Ratio vegetation/ affected surface	Veg/AS	ratio
	Average fire size	FireSize	km^2^
	Fires April to June (fire peak season)	Fires_AtJ	occurrences
	Fires January to March (pre-season)	Fires_JtM	occurrences
	Fires July to December (post-season)	Fires_JtD	occurrences
	Number of severe fires (tree mortality > 50%)	Fires_Sev	occurrences
	Fires attributed to hunting	Fires_Hunt	occurrences
	Fires caused intentionally	Fires_Int	occurrences
	Fires attributed to agriculture	Fires_Agri	occurrences
	Fires by other causes	Fires_Other	occurrences

The formats of the resulting data were highly diverse, including continuous data, such as information regarding elevation, polygons, the extent of different types of vegetation, lines (as in the case of roads), and point data for local communities. A 5x5 km grid allowed it to analyze these data consistently and without bias, following previous research approaches in studying pyrogeographies. The grid cells are about five times the maximum size of wildfires registered in the area between 2010 and 2020 (5.5 km^2^).

Regarding the relation of the pyrogeographic clusters to the current spatial configuration of fire management activities carried out in the reserve, data on fire suppression activities in 2023 were obtained from the regional office for South Mexico of the National Commission of Protected Natural Areas. The information was supplemented with interviews with the person responsible for risk management in this regional office, two of the officials in charge of fire management of the reserve, and two of the regional officials of the National Forestry Commission responsible for the area surrounding the reserve, and the representative of a local non-governmental organization.

### Data selection based on correlation and the variance inflation factor

The study employed a correlation matrix to manage the complexity of the data set, which included a relatively large number of variables. This approach is helpful in identifying redundant variables, thus enhancing the interpretability of the data and simplifying the analysis without significant loss of information [26]. The correlation threshold was established at ±0.9 to ensure only variables with strong similarities were considered equivalent. This criterion allowed it to reduce redundancy effectively by retaining only one variable from each highly correlated pair. The choice of which variable to retain was guided by each variable's relevance to fire dynamics and its contribution to the overall interpretability of the data.

After excluding highly correlated variable pairs using the correlation matrix (r > 0.9), the study applied the Variance Inflation Factor (VIF) test to the remaining variables to diagnose multicollinearity. This method was selected for detecting not just pairwise correlations but more complex relationships among groups of variables [27], ensuring that each variable included in the analysis contributed independently to interpreting pyrogeographical dynamics.

The calculation of VIF values resulted from fitting a linear model, using total burned area as the response variable, given its central role in shaping regional fire dynamics and its relevance in interpreting pyrogeographic patterns. This allowed it to quantify the degree of multicollinearity among predictors. Variables with a VIF exceeding ten were considered highly collinear [28] and were subsequently removed from the data set. This approach guaranteed that the statistical significance of independent variables was not compromised by multicollinearity. Both correlation and VIF analyses were performed using R statistical software (version 4.0.2).

### Hierarchical clustering

The clustering of the grid cells according to their environmental, social and fire-related attributes employed a Ward-like hierarchical clustering algorithm implemented in the ClustGeo R package. While other clustering methods, such as k-means and DBSCAN, were also evaluated, hierarchical clustering was chosen for its robust capability to integrate geographical constraints with pyrogeographic features. Furthermore, the use of a mixing parameter alpha in the ClustGeo algorithm allows it to finely balance the influence of feature space and constraint space [29], tailoring the clustering process to the unique characteristics of the study area. Notably, hierarchical clustering has been effectively employed in similar studies, such as by Conedera and his collaborators, who used it to define areas with similar fire regimes, emphasizing its relevance in fire-related ecological research [5].

In the initial step of the analysis, the 101 grids within the La Sepultura Biosphere Reserve were partitioned into *K* clusters based on a dissimilarity matrix that represented environmental, social, and fire-related variables. After determining the appropriate number of clusters, *K*, four different clustering scenarios were explored, each varying in the degree of spatial constraints applied. First, the resulting areas solely expressed attribute dissimilarity. This attribute-based clustering formed clusters according to similarities in environmental, social, and fire-related variables without contiguity constraints, resulting in clusters that were not necessarily spatially continuous. On the contrary, a second approach with strict spatial adjacency produced clusters that showed minimal internal attribute similarity. Consequently, to achieve cluster that were spatially compact and with low feature dissimilarities, the study subsequently incorporated a matrix of attribute dissimilarities alongside the geographical dissimilarities, applying soft contiguity constraints using a mixing parameter (α) set at 0.2 and 0.3, respectively. In this context, soft contiguity refers to a flexible clustering approach that considers both attribute similarity and geographical proximity without strictly enforcing spatial adjacency [30].

In the ClustGeo algorithm, α adjusts the relative weight between feature dissimilarities (e.g., environmental, social, fire-related variables) and spatial dissimilarities: lower α values prioritize attribute similarity, whereas higher α values promote spatial compactness. This method increases geographical cohesion while preserving the heterogeneity of non-spatial attributes. Unlike hard spatial constraints, which require immediate adjacency, soft contiguity enables the formation of more nuanced and adaptive cluster structures [29].

### Cluster interpretation

After applying hierarchical clustering to delineate distinct groups within the data set, the study aimed to identify the underlying environmental, social, and fire-related drivers distinguishing each cluster. Principal Component Analysis (PCA) was conducted, a classic statistical technique widely used in many research fields, including wildfire modelling [31,32].

PCA is primarily used to reduce the dimensionality of large datasets through orthogonal decomposition, highlighting key patterns by emphasizing the most critical features [33]. Initially, variables were standardized to mean zero and unit variance, ensuring equal weighting in the analysis [34]. The next step was the computation of the covariance matrix to extract eigenvalues and eigenvectors, which revealed the variance captured by each principal component and their directions in the multidimensional space. The contribution of variables to the first two principal components was assessed using cos2 values, with higher values indicating a significant influence on each component. This method enabled it to uncover the most representative environmental, social, and fire-related factors defining each pyrogeographic cluster.

Boxplots were generated for each cluster resulting from the two partitioning approaches (no spatial constraints and neighborhood relationships). These box plots were created for relevant environmental, social, and fire-related variables identified as significant in the PCA analysis. This graphical representation allowed it to visually assess the distribution and range of key variables within each cluster, complementing the PCA findings by highlighting outliers, median values, and variability. Boxplot analyses were performed using R statistical software (version 4.0.2), while PCA was conducted utilizing FactoMineR and factoextra packages within the same software environment.

These analyses were applied to different approaches given to the spatial proximity and cohesiveness of the cluster grids. This included applying neighbourhood constraints. However, even if clusters consisted of areas that shared just a corner point, the result was unsatisfactory. The resulting clusters showed high heterogeneity within the values of the variables of the areas included in the clusters, contrary to the aim to generate pyrogeographic zones characterized by specific characteristics. Therefore, the results do not enforce spacial adjacency.

In addition, the clusters were integrated into a geographic information system in ArcMap and visualized on a map. This representation also included administrative boundaries and the location of fire management actors that work in the area, to compare the identified clusters with the current configuration of fire management.

## Results

### Correlations and multicollinearity

Several variables, such as the number of fires, the number of fires in the dry season, and the number of fires caused by agricultural activities, had to be excluded due to a very high correlation (r > 0.9) with the burned area variable (see Supporting Information for a complete list of the excluded variables). This means that these variables are generally characteristic of a large proportion of the fires in this area, which aligns with previous research [17]. A similarly high correlation existed between population variables. Among variables with multicollinearity (VIF > 10), there were areas covered by fire-adapted vegetation and crops.

Although excluding these variables was crucial to ensure the robustness and independence of the subsequent cluster analysis, their relevance to interpreting the results should not be ignored. For example, it should be assumed that most fires in the La Sepultura Biosphere Reserve originated from agricultural practices and predominantly occurred during the dry season. While most of these very high correlations and multicollinearities were rather unsurprising, the revision of less high but still considerable correlations between variables did show some interesting results regarding some other variables. The correlation between road length and wildfires caused by “other” factors (excluding agricultural burning, hunting, and intentionally caused fires) was 0.59. The study did not have access to any more detailed data to shed light on this situation. One potential explanation could be fires caused by cigarettes thrown from cars driving by; however, more research is needed to clarify this issue. Moreover, a relatively strong correlation (0.53) between burned fire-sensitive vegetation and fires caused by hunters, indicates that fires related to this cause are particularly problematic for forest conservation.

### Cluster analysis

**Fig 3:**
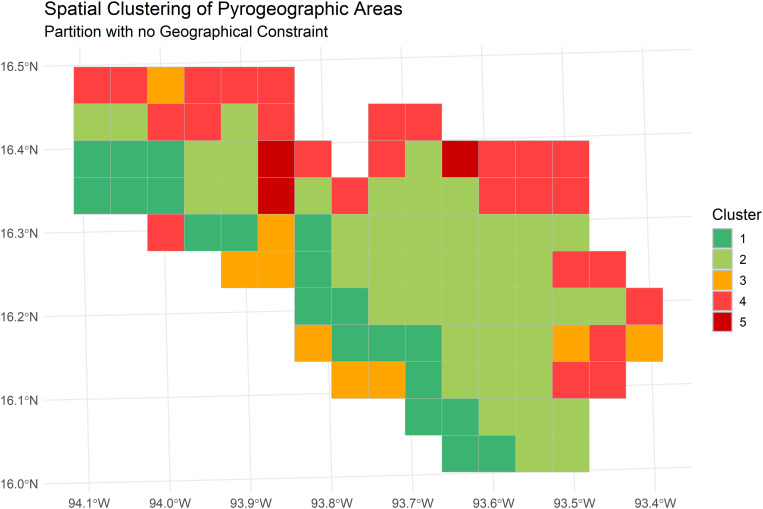
Spatial clusters of pyrogeographic areas within the La Sepultura Biosphere Reserve based on environmental, social, and fire-related attributes, using a Ward-like clustering algorithm.

**Fig 4:**
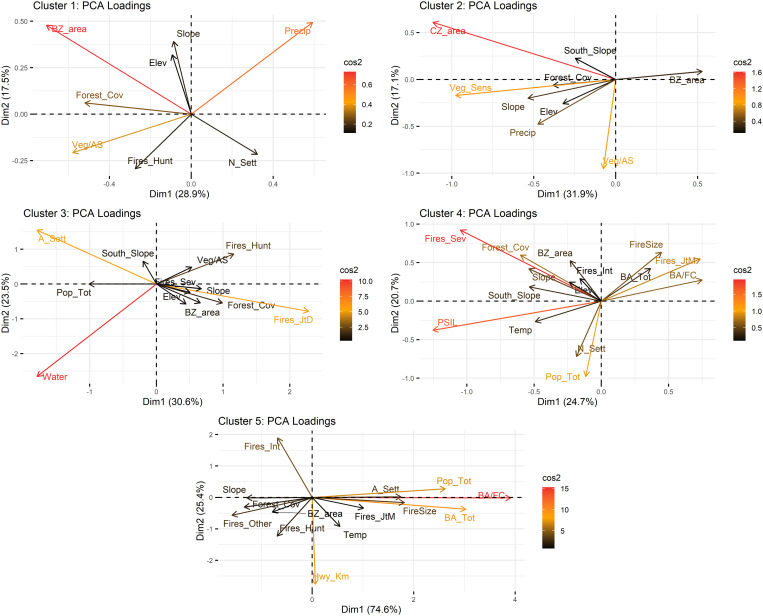
PCA biplots showing variable loadings for the first two principal components across the five pyrogeographic clusters, with vectors representing the direction and strength of each variable’s contribution and Cos² values (visualized as a colour gradient) indicating the quality of representation of each variable. For clarity, only variables with moderate to high cos² contributions were included in each biplot. Full variable names and descriptions are provided in Table 2 of the Methods section.

**Fig 5:**
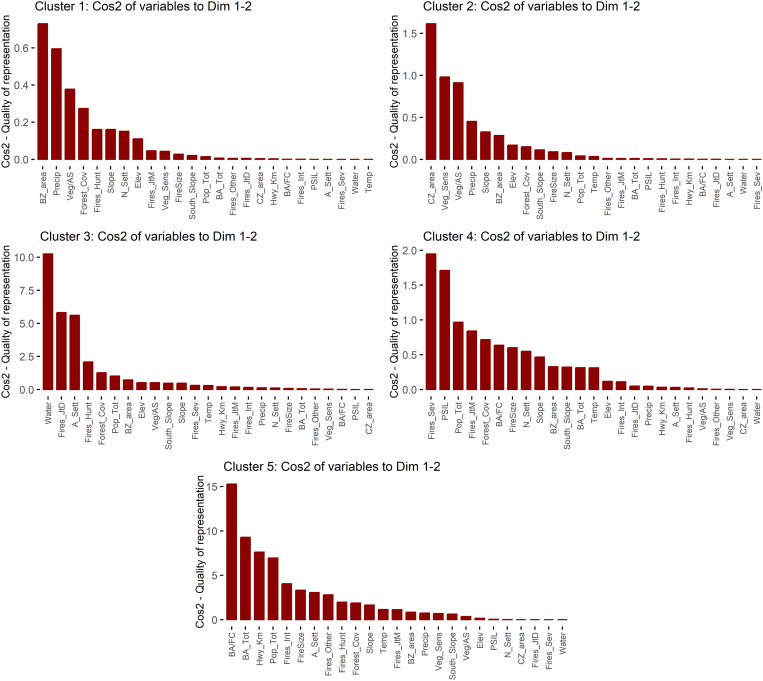
Quality of representation of the variables per cluster (variable codes: see Table 2).

**Fig 6:**
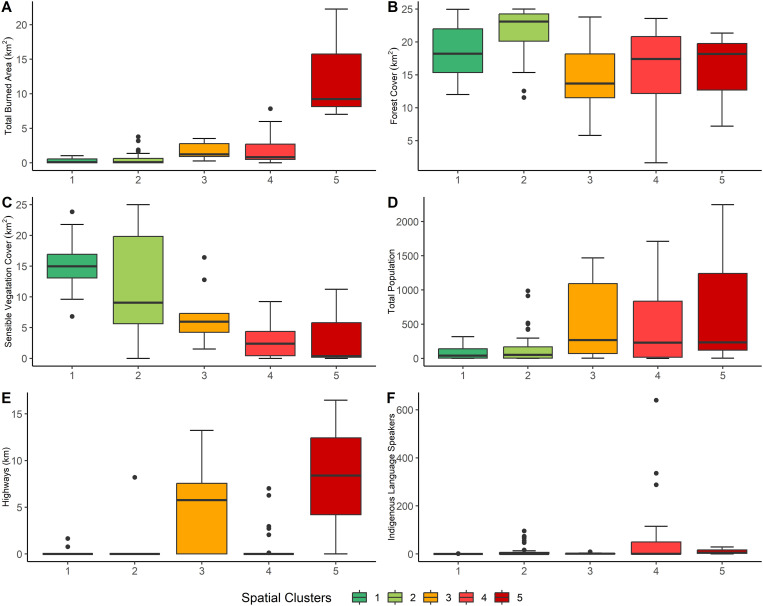
Box plots for selected variables included in the cluster analysis.

As outlined in Chapter 2.2.4, it was impossible to generate internally homogeneous clusters regarding the variables included in the study that were also spatially contiguous. However, the five clusters that resulted from the final analysis do show a relatively clear spatial distribution pattern (Fig 3). The following paragraphs explore the characteristics of each of the clusters, including PCA loadings (see also Fig 4), the quality of representation of the variables per cluster (Fig 5), and the characteristics of the most relevant variables, shown in the Boxplots in Fig 6.

Cluster 1 (20 cells) stretches along the southwestern border of the biosphere reserve but is interrupted in the central part, mainly by cluster 3, which splits the cluster up into two areas that are still connected by a corner point and a further area separated by one cell from the others. The principal component analysis of the cluster indicates that the variables with the major influence are its pertinence to the reserve's buffer zone and environmental variables, including medium annual precipitation, medium slope steepness, and forest area, with an important presence of fire-sensitive vegetation. The proportion of forest areas affected by wildfires is very low, and those that occur are generally small. Human activities are generally of less importance, although among the fires that happen in this area, fires caused by hunters are of relative importance.

Cluster 2 is by far the largest (43 cells) and concentrates the reserve's interior areas (including its core zones); only five of its cells border the outside of the reserve. Natural characteristics dominate, like in cluster 1, including an important presence of fire-sensitive vegetation, a low proportion of burned areas in relation to the total forest area, generally steep slopes, and high precipitation values. On a few occasions, some fires have affected larger forest areas. Population density and other variables related to human activities, such as road length, show very low values. The main difference between this cluster and cluster 1 lies in the fire characteristics: There are no larger fires in cluster 1, but many smaller fires are caused by hunting. Also, the high precipitation values distinguish cluster 2, which means it is even less vulnerable to wildfire than cluster 1.

The primary distribution of Cluster 3 is in the central part of the reserve's southwestern border, with a total of six cells, although there are also three outliers in other parts of the reserve. Cluster 3 comprises several distinct components: the presence of water bodies, the high number of human settlements, the relatively high proportion of fires during the rainy season (July to December), and the considerable number of fires caused by hunting; the latter two may be related as illegal hunting may take place all over the year. Forest areas and total population are further outstanding variables of this cluster (although in terms of population, there are some notable disparities between the areas included in the cluster). Fire incidence is higher than in clusters 1 and 2, and fires tend to be larger, but fire-sensitive vegetation is less prevalent. Furthermore, there is a considerable presence of roads. Compared to cluster 1, which is also located along the southwestern limits of the reserve, there are a few coincidences (high proportion of forest areas, fires caused by hunters) but also determining differences in fire incidence and several population-related variables.

The second largest cluster, cluster 4, is distributed along the reserve's northern, northeastern and eastern limits in three areas (considering connections of corner points). The main variables that characterize this cluster are related to population (total population, presence of population speaking an indigenous language, number of villages) and fire incidence (occurrence of severe fires, fires in the early fire season from January to March, proportion between forest area and burned area, and medium fire size). In contrast to clusters 1 and 2 and like cluster 3, this cluster represents an area highly impacted by human activities. No specific fire cause was found for this cluster, suggesting agricultural burning as the predominant cause. Together, cluster 3 and cluster 4 indicate the role of spatial proximity to human settlements in an area's fire incidence.

Regarding fire size, the boxplots reveal some outliers that point to large fires registered in a few areas. What is striking is the difference between this cluster at the northern and cluster 2 along the reserve's southern limits, which also shows in the absence of fire-sensitive vegetation in cluster 4. Another relevant observation in this cluster is the low number of road kilometers, considering its relatively high population density, possibly indicating the presence of highly marginalized communities.

Cluster 5 is the smallest one, with three cells, two of which are directly connected; all are located on the northern central part of the reserve, near the reserve's boundaries. Moreover, it stands out for its uniquely high burned area values and the high proportion of burned areas in relation to the total forest area, together with the medium size of fires. All areas are near the reserve's boundaries, although they also contain a small part of the core zones.

### Relation of the clusters to fire management activities

**Fig 7:**
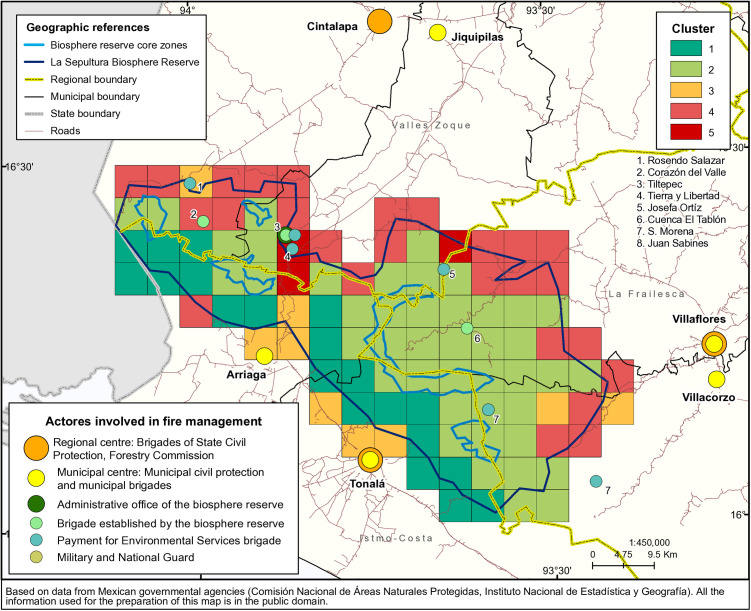
Spatial configuration of fire management activities (data derived from Mexican governmental agencies [21,23,35], and data provided by the regional directorate of the CONANP (National Commission of Protected Natural Areas); all information in the figure is in the public domain).

Fig 7 puts the clusters – with colours adapted to their respective fire behaviour – in relation to the spatial configuration of the actors involved in fire management in the La Sepultura biosphere reserve, based on data from Mexican governmental authorities [21,23,35]. These actors operate at different spatial levels. At the highest level are three regions: Valles Zoque, Frailesca, and Istmo-Costa. In each of these regions, one town concentrates the leading regional actors involved in fire management, the regional centres of the National Forestry Commission and of the Secretary of Civil Protection of the State of Chiapas. In Cintalapa, the main town of the Valles Zoque region, there is also an encampment of the Mexican military and the National Guard, which sometimes help with firefighting, mainly within the Valles Zoque region. In Frailesca, the only relevant non-governmental organization, Biomasa, supports community-based fire management plans and prescribed burning along the western part of the reserve.

The next lower level is the municipal level, with six municipalities; the main actors, the municipal civil protection bodies and temporary brigades financed by the National Forestry Commission, have their centres in the municipal head towns. None of these head towns lies within the biosphere reserve, but road connections usually allow it to reach the reserve's limits in about half an hour; transport within the reserve is more difficult, and only one road crosses the reserve from north to south. Right on this road is the biosphere reserve's administrative office, managed by the National Commission of Protected Areas. One of the temporary brigades established by this commission is in the same place. Another one is found in the central part of the northeastern area of the reserve, and another one in the central western part. The lowest level is that of local communities, which have established brigades that take care of their community land, with financial support via a payment for environmental services program set up by the National Forestry Commission.

Most of these actors are mainly focused on fire suppression, with small physical prevention goals (a few kilometers per year of fire breaks that are maintained annually) and some cultural prevention events, mostly talks at community meetings. Some, like the military, the National Guard and all actors related to civil protection (state and municipal), are almost entirely suppression-focused. Prescribed burning is only practiced on a very small scale at experimental plots, with the participation of the organization Biomasa, the reserve administration, and local communities. There are almost no efforts regarding legal fire prevention.

The distribution of the clusters aligns in several ways with the outline of the management activities in the area. Clusters 1 and 2 almost entirely belong to the Istmo-Costa region, which could permit a homogeneous fire management approach in this area. Cluster 4 splits between the Valles Zoque and Frailesca regions; the location of these more critical areas at the outer limits of the reserve makes them relatively easy to reach from the municipal head towns and the regional centres. Valles Zoque also holds two of the cells of cluster 5, while the other one is situated at the limits with Frailesca. The two connected cells of cluster 5 are the area that receives the most attention, with three brigades within the area and at the road that connects to the nearest regional centre. Cluster 2 lies in the central area, split between different regions and relatively far from most actors, except for some temporal community brigades.

Several management implications arise from analyzing the clusters' distribution and the characteristics described in the previous section. The management of the area belonging to cluster 1, confined to the Istmo-Costa region, seems to work relatively well despite being far away from most of the main actors involved in fire management. However, several cells share a border with cells from cluster 3 or even cluster 4. In these cases, fire breaks could diminish the risk of fire spread, especially in very dry years, to protect the area's fire-sensitive vegetation.

In cluster 3, an area of relatively high fire incidence, the local actors are almost entirely suppression-focused. Thus, it is necessary that some of these actors take up prevention tasks, too, or that actors working in prevention from other parts step in.

Clusters 1 and 3 together concentrate the fires caused by illegal hunting. Thus, this area requires an integral strategy to reduce the fires caused by this activity. Cluster 2 seems relatively well-protected from adverse fire effects. However, there have been some larger fires, related to problems of accessibility and the distance of most of the cells of the cluster from the places where the main fire management actors are. In this sense, it would be beneficial to consider the establishment of several additional community-based brigades, especially in the large eastern part, to safeguard this area, also by physical prevention measures, especially where they share a border with the cells of clusters 3, 4, and 5.

Cluster 4 and especially cluster 5 are the areas with the highest fire intensity. However, they are also, for the most part, relatively well attended by fire management actors, particularly the area with two cells of cluster 5 in the northwestern part of the reserve that concentrates the location of several brigades. However, the other cluster 5 cell and a part of the cluster 4 cells at the northeastern limit of the reserve are relatively far away from any of the main actors; here, the establishment of at least one more brigade nearby and prevention activities where actors from less close places could come in to help might prove beneficial.

## Discussion

The present study showed that pyrogeographic zonation is a method that can be applied at the local level, resulting in clearly defined, albeit in this case not contiguous, zones that are relevant to understanding an area's fire dynamics and that can inform fire management strategies. It is essential to state that the resulting clusters are by no means the only way to apply a pyrogeographic zonation at the local level, as the different approaches outlined in the Introduction demonstrate, such as Kernel density estimation [9] or Random Forests Modelling [8,16]. However the study applied a hierarchical cluster analysis approach [5], which allows the integration of a variety of data and a detailed interpretation of the configuration of the clusters. The following paragraphs discuss the main issues identified during the different stages of this study.

### Selection of the spatial units and datasets

Regarding the definition of spatial units, studies focusing on larger areas take advantage of predefined regionalization, such as statistical and administrative regions, such as the NUTS regions in Europe [5,6] or ecoregions [13,14]. In the present case, no such delimitation could be identified. This issue was solved using a grid, following previous approaches [7,8,15],. The size of the grid cells is crucial in this regard; too large ones might include very heterogeneous areas, while too small ones could bring about a degree of complexity that would make interpretation much more difficult. In the present case, the grid size has been adequate, given the definition of clearly defined clusters. In other study areas and at other spatial levels, these units must be adapted to the specific spatial context.

The type of data used is of crucial importance in pyrogeographic clustering. While some approaches follow a more simplified procedure, this paper aligns with previous studies that aim at a data set as complete as possible in terms of variables that might be related to an area's pyrogeography [5,6,8]. Nevertheless, the data selection is not the same as in these studies, depending on the data sources available for the selected study area and the spatial scope. Some of the variables used in other studies could not be included here, yet it was the first of its kind to consider fire causes.

Including different variables gives room for a discussion on which of them is most defining of the resulting clusters. For Conedera and collaborators [5], the main determining factors identified by the study were population density, fire-prone Mediterranean vegetation and climatic factors, including wind. Elia and collaborators also identified climate (temperature and exposure to droughts) and population density as the central defining factors [6]. The present study is aligned with these results, as the presence of human settlements is an apparent defining factor of the clusters. Also, the presence of natural vegetation and its relationship to fire were found to be of importance. The density of roads and the causes of fire were important aspects identified here that previous studies had not highlighted.

One aspect that was of surprising low importance, compared to previous studies in this field, was climate. Only in one of the clusters, precipitation was a defining element. The main explanation for this is the low number and degree of detail of the available official climate data that could be used for a study at this spatial scale in Mexico. In general, climate types might be of less importance in a study area of the size of La Sepultura. Nevertheless, it is important to consider this as an limitation when interpreting the results and applying them to fire management planning as discussed in chapter 4.2. The lack of more detailed climate data also compromises the opportunity to realize predictive analysis or the creation of scenarios in the context of climate change. One way this might be addressed could be by integrating climate data in grid format from global reanalysis data [36].

### Implications of the zonation results

Several previous studies used zonation results for subsequent analyses regarding the potential alterations of pyrogeographic zones under a changing climate [15,16]. This might be undertaken in the present case, too, as two climate aspects, temperature and precipitation, were considered in the analysis. However, the present study's focus concerned a different issue: the relevance of the zones for fire management.

Most studies state their results are critical for fire management, but this is generally only framed in generalized statements without any more concrete recommendations. The overall argument is that by knowing an area's zonation in this regard, human and material resources can be better allocated, and necessary preventive measures can be applied in a more focused way. This study proposed to advance further in this context. To do so, it put the pyrogeographic zones in relation to the current configuration of fire management measures, which implied the combination of very different data sources, both quantitative and qualitative; mapping provided an adequate tool to integrate this information, as had been shown in previous studies regarding the relation of fire management and an area's environmental conditions [37,38].

However, this should be seen as the first steps into this direction; future studies might directly explore the inclusion of pyrogeographic zonation in fire management strategies to see how relevant they are in practice. First, this implies an effective communication of the results, reaching relevant stakeholders, and potentially the involvement of researchers in the planning process of the actors working in fire management. Previous research has shown that fire management professionals generally have been open to incorporating new inputs from the academia, but this knowledge transfer has been slow and there exist distinct barriers and limitations. For example, the complexity of academic results and their presentation in academic language, information overload, and the nonalignment of timing of research and decision making in practice [39–41]. Thus, several measures need to be taken to facilitate this knowledge transfer, such as the integration of scientists into planning panels, the involvement of people with experience in both science and practice who can act as boundary spanners. Moreover, the publication and distribution of information using diverse kinds of media and being presented in a way directly focused on and using the technical language of fire management practitioners [40,42].

In the case of the present study, the clustering approach without spatial adjacency brought about results with a degree of complexity that might be a barrier to direct application. In this sense, the results should be mainly seen as a starting point for a dialogue regarding an enhanced spatial distribution and zoning of fire management activities, rather than fixed recommendations. Beside the attribute similarities highlighted in the clustering approach, this dialogue might include the expertise of local fire managers and practical issues such as accessibility and the benefits of contiguous working areas.

It is important to note that current management efforts follow predefined political boundaries. Thus, implementation of management measures based on this research might only be carried out partially in some of the reserve’s areas. Transcending municipal and regional boundaries is challenging and requires coordination and openness from all the involved administrative authorities, which highlights the importance of multi-scale governance in fire management [43,44]. It would also be interesting to include the perspectives of local actors from the outset in establishing the pyrogeographic clusters by methods such as participatory mapping [45]. This way, pyrogeographic zonation could be integrated as a tool for novel fire management approaches, such as intercultural fire management [46].

## Conclusion

Pyrogeographic zonation is an approach to define areas with similar characteristics in terms of the role of fire in the landscape and the different factors that influence fire occurrence. The first publications in this field date from over ten years ago, yet it has gained momentum in recent years. The present study follows previous approaches and adapts them to the local level, focusing on the case of the La Sepultura Biosphere Reserve. The different variables considered – especially data related to population, vegetation, roads, and fire causes – resulted in five clearly defined zones, which are not entirely contiguous but show a distinct spatial distribution. A further advance in this research is the integration of the zones with a consideration of current fire management patterns, which allows it to arrive at concrete recommendations that better adapt this management to the area's pyrogeography.

## Supporting information

S1 Fig Correlation Matrix of Original Variables (Variables with a pairwise correlation coefficient greater than ±0.9 were considered highly correlated; to reduce redundancy, only one variable from each highly correlated pair was retained for subsequent analyses). (TIF)

S2 Table Results of the Variance Inflation Factor (VIF) analysis following initial pairwise correlation filtering. (PDF)

S3 Table Variables excluded from the analysis due to redundancy or high multicollinearity. (PDF)

## References

[1] Bowman DMJS, O’Brien JA, Goldammer JG. Pyrogeography and the Global Quest for Sustainable Fire Management. Annu Rev Environ Resour. 2013;38(1):57–80. doi: 10.1146/annurev-environ-082212-134049

[2] Rodríguez-Trejo DA. Incendios de vegetación: su ecología. manejo e historia. Mexico City: Colegio de Postgraduados; 2014.

[3] Marlon JR, Bartlein PJ, Carcaillet C, Gavin DG, Harrison SP, Higuera PE, et al. Climate and human influences on global biomass burning over the past two millennia. Nat Geosci. 2008;1: 697–702. doi: 10.1038/ngeo313

[4] Pyne SJ. Fire: A short history. 2nd ed. Seattle: University of Washington Press; 2019.

[5] Conedera M, Krebs P, Valese E, Cocca G, Schunk C, Menzel A, et al. Characterizing Alpine pyrogeography from fire statistics. Applied Geography. 2018;98:87–99. doi: 10.1016/j.apgeog.2018.07.011

[6] Elia M, Giannico V, Ascoli D, Argañaraz JP, D’Este M, Spano G, et al. Uncovering current pyroregions in Italy using wildfire metrics. Ecol Process. 2022;11(1). doi: 10.1186/s13717-022-00360-6

[7] Moreno MV, Chuvieco E. Characterising fire regimes in Spain from fire statistics. Int J Wildland Fire. 2013;22(3):296. doi: 10.1071/wf12061

[8] Boulanger Y, Gauthier S, Gray DR, Le Goff H, Lefort P, Morissette J. Fire regime zonation under current and future climate over eastern Canada. Ecol Appl. 2013;23(4):904–23. doi: 10.1890/12-0698.1 23865239

[9] Wu Z, He HS, Yang J, Liang Y. Defining fire environment zones in the boreal forests of northeastern China. Sci Total Environ. 2015;518–519:106–16. doi: 10.1016/j.scitotenv.2015.02.063 25747370

[10] Trigo RM, Sousa PM, Pereira MG, Rasilla D, Gouveia CM. Modelling wildfire activity in Iberia with different atmospheric circulation weather types. Intl Journal of Climatology. 2013;36(7):2761–78. doi: 10.1002/joc.3749

[11] Calheiros T, Nunes JP, Pereira MG. Recent evolution of spatial and temporal patterns of burnt areas and fire weather risk in the Iberian Peninsula. Agricultural and Forest Meteorology. 2020;287:107923. doi: 10.1016/j.agrformet.2020.107923

[12] Calheiros T, Pereira MG, Nunes JP. Assessing impacts of future climate change on extreme fire weather and pyro-regions in Iberian Peninsula. Sci Total Environ. 2021;754:142233. doi: 10.1016/j.scitotenv.2020.142233 32920419

[13] Resco de Dios V, Cunill Camprubí À, Pérez-Zanón N, Peña JC, Martínez Del Castillo E, Rodrigues M, et al. Convergence in critical fuel moisture and fire weather thresholds associated with fire activity in the pyroregions of Mediterranean Europe. Sci Total Environ. 2022;806(Pt 4):151462. doi: 10.1016/j.scitotenv.2021.151462 34742803

[14] Resco de Dios V, Cunill Camprubí À, He Y, Han Y, Yao Y. North-south antiphase of wildfire activity across the pyroregions of continental China driven by NAO and the Antarctic oscillation. Sci Total Environ. 2023;859(Pt 2):160386. doi: 10.1016/j.scitotenv.2022.160386 36427739

[15] Galizia LF, Barbero R, Rodrigues M, Ruffault J, Pimont F, Curt T. Global Warming Reshapes European Pyroregions. Earth’s Future. 2023;11(5). doi: 10.1029/2022ef003182

[16] Cunningham CX, Williamson GJ, Nolan RH, Teckentrup L, Boer MM, Bowman DMJS. Pyrogeography in flux: Reorganization of Australian fire regimes in a hotter world. Glob Chang Biol. 2024;30(1):e17130. doi: 10.1111/gcb.17130 38273509

[17] Huffman MR Community-based fire management at La Sepultura Biosphere Reserve, Chiapas, Mexico. Doctoral thesis, Colorado State University. 2010. https://mountainscholar.org/items/f5a9142d-dc19-4a07-9975-fce66fdc0e11

[18] Marín P-G, Julio CJ, Dante Arturo R-T, Daniel Jose V-N. Drought and Spatiotemporal Variability of Forest Fires Across Mexico. Chin Geogr Sci. 2018;28(1):25–37. doi: 10.1007/s11769-017-0928-0

[19] Instituto Nacional de Ecología. Programa de manejo. Reserva de la biosfera La Sepultura. Mexico City (Mexico): Instituto Nacional de Ecología; 1999.

[20] Natural Earth. Downloads Database. Internet. 2024 [cited 2024 Sep 20]. https://www.naturalearthdata.com/downloads/

[21] Comisión Nacional de Áreas Naturales Protegidas. Información espacial de las áreas naturales protegidas. Database Internet. 2024 [cited 2024 Sep 20]. https://sig.conanp.gob.mx/Shape

[22] Instituto Nacional de Estadística y Geografía. Continuo de Elevaciones Mexicano 3.0 (CEM 3.0). Database Internet. 2020 [cited 2025 May 30]. https://www.inegi.org.mx/app/geo2/elevacionesmex/

[23] Instituto Nacional de Geografía y Estadística. Marco Geoestadístico 2024. Conjunto de datos vectoriales de entidades federativas, municipios y localidades, escala 1:250 000; 2024 [cited 2025 May 30]. https://www.inegi.org.mx/app/biblioteca/ficha.html?upc=794551132173

[24] López J. Mapa de regiones ambientales biofísicas de México (NA XV), escala 1:4000000. In: Coll-Hurtado A, coordinator. Nuevo Atlas Nacional de México. Mexico City (Mexico): Instituto de Geografía, National Autonomous University of Mexico; 2007.

[25] Instituto Nacional de Geografía y Estadística. Censo de Población y Vivienda 2020 Database Internet.; 2021 [cited 2024 Oct 10]. https://www.inegi.org.mx/programas/ccpv/2020/

[26] Al‐Kandari NM, Jolliffe IT. Variable selection and interpretation in correlation principal components. Environmetrics. 2005;16(6):659–72. doi: 10.1002/env.728

[27] Oke J, Akinkunmi WB, Etebefia SO. Use of correlation, tolerance and variance inflation factor for multicollinearity test. Global Scientific Journal. 2019;7(5):652–9.

[28] Vittinghoff E, Glidden DV, Shiboski SC, McCulloch CE. Regression methods in biostatistics: linear, logistic, survival, and repeated measures models. Berlin: Springer Science & Business Media; 2012.

[29] Chavent M, Kuentz-Simonet V, Labenne A, Saracco J. ClustGeo: an R package for hierarchical clustering with spatial constraints. Comput Stat. 2018;33(4):1799–822. doi: 10.1007/s00180-018-0791-1

[30] Bourgault G, Marcotte D, Legendre P. The multivariate (co)variogram as a spatial weighting function in classification methods. Math Geol. 1992;24(5):463–78. doi: 10.1007/bf00890530

[31] Xu D, Shao G, Dai L, Hao Z, Tang L, Wang H. Mapping forest fire risk zones with spatial data and principal component analysis. SCI CHINA SER E. 2006;49(S1):140–9. doi: 10.1007/s11434-006-8115-1

[32] Jiménez-Ruano A, Mimbrero MR, de la Riva Fernández J. Understanding wildfires in mainland Spain. A comprehensive analysis of fire regime features in a climate-human context. Applied Geography. 2017;89:100–11. doi: 10.1016/j.apgeog.2017.10.007

[33] Li S, Banerjee T. Spatial and temporal pattern of wildfires in California from 2000 to 2019. Sci Rep. 2021;11(1):8779. doi: 10.1038/s41598-021-88131-9 33888784 PMC8062671

[34] Mardia KV, Kent JT, Bibby JM. Multivariate analysis. London (UK): Academic Press; 1979.

[35] Instituto Nacional de Geografía y Estadística. Red Vial. Red Nacional de Caminos (RNC); Database Internet. 2023 [cited 2025 May 30]. https://www.inegi.org.mx/app/biblioteca/ficha.html?upc=794551067307

[36] Hersbach H, Bell B, Berrisford P, Hirahara S, Horányi A, Muñoz‐Sabater J, et al. The ERA5 global reanalysis. Quart J Royal Meteoro Soc. 2020;146(730):1999–2049. doi: 10.1002/qj.3803

[37] Hamilton M, Fischer AP, Ager A. A social-ecological network approach for understanding wildfire risk governance. Global Environmental Change. 2019;54:113–23. doi: 10.1016/j.gloenvcha.2018.11.007

[38] Neger C, Evers CR, Romero Cuapio O, Páramo Gómez J de D. Integrating social network analysis and cartography: the case of fire management in a Mexican biosphere reserve. International Journal of Cartography. 2024;:1–19. doi: 10.1080/23729333.2024.2392213

[39] Calkin DC, Finney MA, Ager AA, Thompson MP, Gebert KM. Progress towards and barriers to implementation of a risk framework for US federal wildland fire policy and decision making. Forest Policy and Economics. 2011;13(5):378–89. doi: 10.1016/j.forpol.2011.02.007

[40] Hunter ME. Outcomes of fire research: is science used?. Int J Wildland Fire. 2016;25(5):495. doi: 10.1071/wf15202

[41] McFayden CB, Johnston LM, Woolford DG, George C, Boychuk D, Johnston D, et al. A Conceptual Framework for Knowledge Exchange in a Wildland Fire Research and Practice Context. Studies in Big Data. Springer International Publishing. 2023. p. 165–84. doi: 10.1007/978-3-031-29937-7_12

[42] Adams T “Ted,” Butler BW, Brown S, Wright V, Black A. Bridging the divide between fire safety research and fighting fire safely: how do we convey research innovation to contribute more effectively to wildland firefighter safety? Int J Wildland Fire. 2017;26(2):107. doi: 10.1071/wf16147

[43] Hamilton M, Nielsen-Pincus M, Evers C. Wildfire risk governance from the bottom up: linking local planning processes in fragmented landscapes. E&S. 2023;28(3). doi: 10.5751/es-13856-280303

[44] Neger C, Monzón‐Alvarado CM, Guibrunet L. Fire governance research in the tropics: A configurative review and outline of a research agenda. Singap J Trop Geogr. 2024;45(2):332–46. doi: 10.1111/sjtg.12534

[45] Haworth B, Whittaker J, Bruce E. Assessing the application and value of participatory mapping for community bushfire preparation. Applied Geography. 2016;76:115–27. doi: 10.1016/j.apgeog.2016.09.019

[46] Bilbao B, Mistry J, Millán A, Berardi A. Sharing Multiple Perspectives on Burning: Towards a Participatory and Intercultural Fire Management Policy in Venezuela, Brazil, and Guyana. Fire. 2019;2(3):39. doi: 10.3390/fire2030039

