# Supplementary figures and images for: Pyrogeographic zonation: Implications for fire management at the local level
Source: PLoS One. 2025 Aug 4;20(8):e0328233. doi: 10.1371/journal.pone.0328233 (PMC12321100; doi:10.1371/journal.pone.0328233)

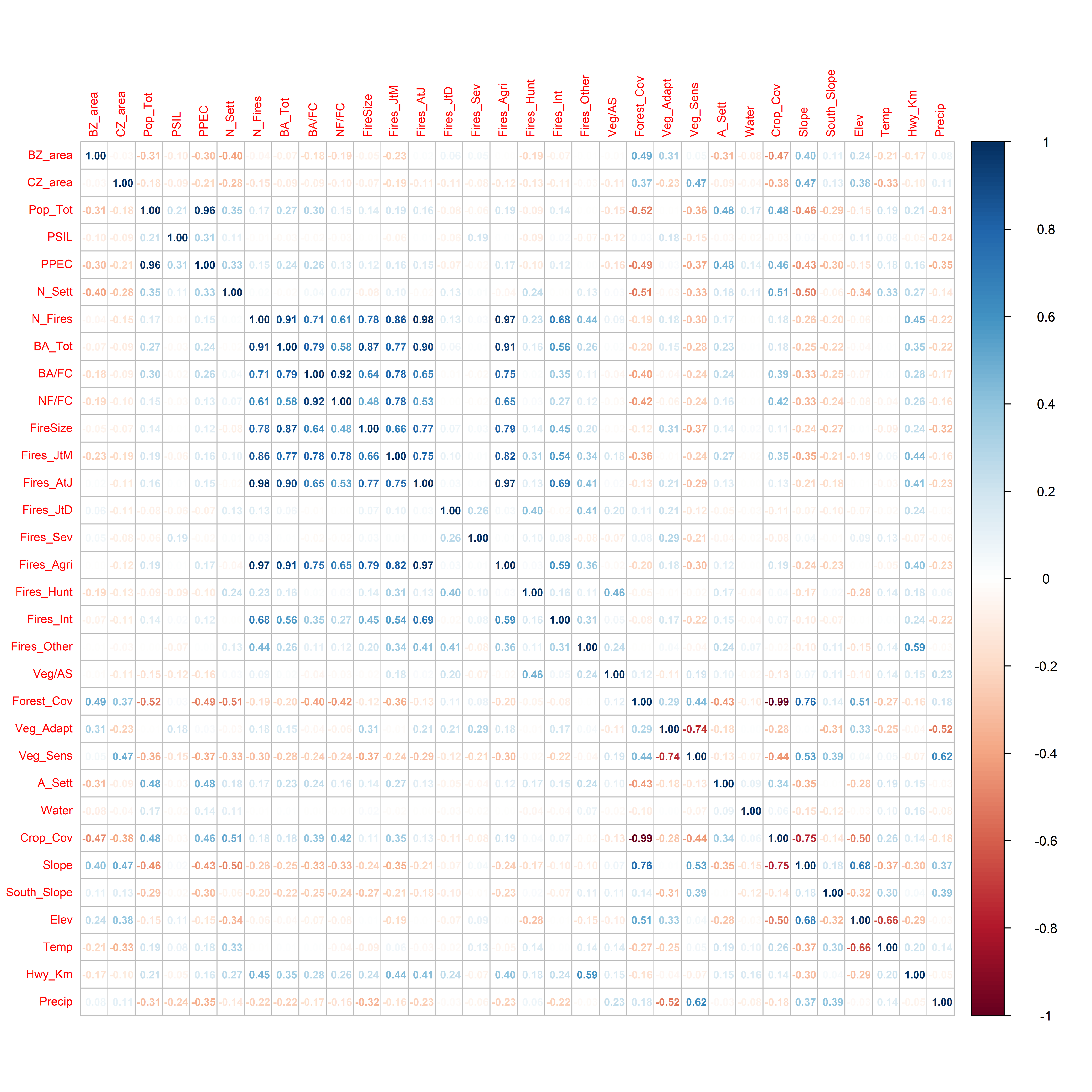

Supplement: S1 Fig — (TIF) [file pone.0328233.s001.tif]
