# Supplementary material for: Pyrogeographic zonation: Implications for fire management at the local level
Source: PLoS One. 2025 Aug 4;20(8):e0328233. doi: 10.1371/journal.pone.0328233 (PMC12321100; doi:10.1371/journal.pone.0328233)
Supplement: S2 Table — (PDF) [file pone.0328233.s002.pdf]

S2 Table. *Results of the Variance Inflation Factor (VIF) analysis following initial pairwise correlation filtering. Note: Variables with VIF values greater than 10 were excluded to reduce multicollinearity, retaining only one variable from each highly collinear group. A linear model was fitted using total burned area as the response variable to calculate VIF values.*

**Variable**	**VIF**	**Variable**	**VIF**
BZ_area	1.99	Fires_Other	3.35
CZ_area	2.19	Veg/AS	1.65
Pop_Tot	2.60	Forest_Cov	4.93
PSIL	1.36	Veg_Sens	22.61
N_Sett	2.20	Veg_Adapt	21.98
BA/FC	4.95	A_Sett	2.01
FireSize	2.69	Water	1.15
Fires_JtM	6.40	Slope	6.66
Fires_JtD	2.68	South_Slope	2.04
Fires_Sev	1.58	Elev	6.09
Fires_Hunt	3.53	Temp	2.85
Fires_Int	1.85	Precip	2.31
		Hwy_Km	3.39
