# Supplementary material for: Pyrogeographic zonation: Implications for fire management at the local level
Source: PLoS One. 2025 Aug 4;20(8):e0328233. doi: 10.1371/journal.pone.0328233 (PMC12321100; doi:10.1371/journal.pone.0328233)
Supplement: S3 Table — (PDF) [file pone.0328233.s003.pdf]

S3 Table. *Variables excluded from the analysis due to redundancy or high multicollinearity.*
Note: Based on pairwise correlation (r > 0.9) and Variance Inflation Factor (VIF > 10) tests.

Variable Name	Variable Description	Reason for Exlusion
PPEC	Population with Primary Education Completed	Correlated with Total Population (r = 0.96)
N_Fires	Number of Fires	Correlated with Total Burned Area (r = 0.91)
NF/FC	Number of Fires-to-Forest Cover Ratio	Correlated with Burned Forest Ratio (r = 0.92)
Fires_Agric	Fires Attributed to Agricultural Activities	Correlated with Total Burned Area (r = 0.91)
Fires_AtJ	Fires Recorded from April to June	Correlated with Total Burned Area (r = 0.90)
Crop_Cov	Area Covered by Crops	Correlated with Forest Cover (r = -0.99)
Veg_Adapt	Fire-Adapted Vegetation	VIF > 10, indicating multicollinearity with Fire-Sensitive Vegetation
